# Epigenetic DNA modifications and vitamin C in prostate cancer and benign prostatic hyperplasia: Exploring similarities, disparities, and pathogenic implications

**DOI:** 10.1016/j.neo.2024.101079

**Published:** 2024-10-29

**Authors:** Jolanta Guz, Ewelina Zarakowska, Pawel Mijewski, Aleksandra Wasilow, Fabian Lesniewski, Marek Foksinski, Bartosz Brzoszczyk, Piotr Jarzemski, Daniel Gackowski, Ryszard Olinski

**Affiliations:** a Department of Clinical Biochemistry, Faculty of Pharmacy, Collegium Medicum in Bydgoszcz, Nicolaus Copernicus University in Toruń, Bydgoszcz 85-092, Poland; b Department of Urology, Jan Biziel University Hospital, Bydgoszcz 85-168; Nicolaus Copernicus University in Toruń, Collegium Medicum in Bydgoszcz, Poland

**Keywords:** Epigenetic DNA modifications, Benign prostatic hyperplasia, Prostate cancer, Vitamin C, TET enzymes

## Abstract

**Objectives:**

Benign Prostatic Hyperplasia (BPH) and Prostate Cancer (PC) are very common pathologies among aging men. Both disorders involve aberrant cell division and differentiation within the prostate gland. However, the direct link between these two disorders still remains controversial. A plethora of works have demonstrated that inflammation is a major causative factor in both pathologies. Another key factor involved in PC development is DNA methylation and hydroxymethylation.

**Methods:**

A broad spectrum of parameters, including epigenetic DNA modifications and 8-oxo-7,8-dihydro-2′-deoxyguanosine, was analyzed by two-dimensional ultraperformance liquid chromatography with tandem mass spectrometry in tissues of BPH, PC, and marginal one, as well as in leukocytes of the patients and the control group. In the same material, the expression of *TETs* and *TDG* genes was measured using RT-qPCR. Additionally, vitamin C was quantified in the blood plasma and within cells (leukocytes and prostate tissues).

**Results:**

Unique patterns of DNA modifications and intracellular vitamin C (iVC) in BPH and PC tissues, as well as in leukocytes, were found in comparison with control samples. The majority of the alterations were more pronounced in leukocytes than in the prostate tissues.

**Conclusions:**

Characteristic DNA methylation/hydroxymethylation and iVC profiles have been observed in both PC and BPH, suggesting potential shared molecular pathways between the two conditions. As a fraction of leukocytes may be recruited to the pathological tissues and can migrate back into the circulation/blood, the observed alterations in leukocytes may reflect dynamic changes associated with the PC development, suggesting their potential utility as early markers of prostate cancer development.

## Highlights

• Characteristic DNA methylation/hydroxymethylation changes have been observed in both PC and BPH, suggesting potential shared molecular pathways between the two conditions.
• Diminished levels of 5-hydroxymethylcytosine may extend beyond tumor tissue to include surrogate tissues like leukocytes, indicating systemic alterations in epigenetic modifications during tumor progression.
• Factors such as chronic inflammation, vitamin C availability, and oxidative stress can influence DNA modification processes, including DNA demethylation mediated by TET enzymes.
• Leukocytes exhibit characteristic epigenetic modification patterns in individuals with BPH and PC, suggesting their potential utility as early markers of prostate cancer development.

## Introduction

Benign Prostatic Hyperplasia (BPH) and Prostate Cancer (PC) are highly prevalent urological conditions affecting elderly males. Both disorders involve aberrant cell division and differentiation within the prostate gland [1]. While BPH typically lacks definitive signs of neoplastic features such as genomic alterations, coding mutations, and copy number alterations [2], some studies indicate a higher number of single nucleotide variants in BPH tissue compared to normal tissue [3]. Moreover, it has been demonstrated that cells from BPH, in contrast to cells from normal prostatic tissue, can induce the growth of prostatic epithelium *in vivo* [3]. This observation aligns with studies suggesting a connection between BPH and PC [4,5].

Cytosine methylated at the fifth position is the principal epigenetic mark regulating gene expression. The presence of this DNA modification in the promoter regions of genes is associated with gene repression, which has a profound impact on cellular identity [6]. Active DNA demethylation, which reverses DNA methylation and activates previously silenced genes, hinges on the molecular machinery involving ten-eleven translocation (TET) proteins. These enzymes catalyze iterative oxidation of 5-methylcytosine (5-mCyt) to 5-hydroxymethylcytosine (5-hmCyt), further to 5-formylcytosine (5-fCyt), ultimately yielding 5-carboxycytosine (5-caCyt) [7]. Finally, the base excision repair (BER) pathway is activated due to thymine DNA glycosylase (TDG) action, enabling the excision and replacement of 5-fCyt and 5-caCyt with cytosine, thereby facilitating DNA demethylation. Experimental findings have revealed that TETs also oxidize thymine to 5-hydroxymethyluracil (5-hmUra), another DNA modification with epigenetic implications [8].

For years, the meaning of vitamin C (VC, ascorbate) in cancer prevention has been pointed out through its potential to neutralize pro-carcinogenic reactive oxygen species (ROS). However, at high, pharmacological (millimolar) concentrations, VC demonstrates pro-oxidant activity in the presence of transition metal ions by providing electrons needed to reduce O_2_ to the superoxide radical, which can be converted to hydrogen peroxide (H_2_O_2_) [9]. Our cell culture study showed that supplementing medium with VC at physiological concentrations (about 100 µM) resulted in approximately 1 mM VC inside the cells and led to a significant increase in the level of epigenetic DNA modifications [10]. The level of 8-oxo-7,8-dihydro-2′-deoxyguanosine (8-oxodG) in DNA may inform about oxidative stress in cells [11]. Although it is difficult to decisively categorize the role of 8-oxodG, it is possible that a small fraction of the 8-oxodG pool may play an epigenetic role [12]. Anyway, both properties of 8-oxodG, such as the damage and the epigenetic mark, may potentially be linked to the homeostasis of prostate cells (for further explanation, see our recent paper [13]).

In numerous diseases, obtaining affected tissues may not be easy, nevertheless, some studies showed that analyzing surrogate tissues (including blood cells) can yield equally informative outcomes (reviewed in [14]). Peripheral blood leukocytes often serve as easily accessible cells in a minimally invasive manner, potentially providing information about DNA modifications in other tissues [15]. Therefore, in the present study, a broad spectrum of parameters capable of influencing the metabolic state of DNA modifications was analyzed for the first time in tissues of benign prostatic hyperplasia, prostate cancer, normal prostate tissue, as well as leukocytes from patients and the control group. In DNA isolated from leukocytes and the prostate tissues, several epigenetic modifications were quantified: 5-methyl-2′-deoxycytidine (5-mdC), 5-(hydroxymethyl)-2′-deoxycytidine (5-hmdC), 5-formyl-2′-deoxycytidine (5-fdC), 5-carboxy-2′-deoxycytidine (5-cadC) and 5-(hydroxymethyl)-2′-deoxyuridine (5-hmdU) and an established oxidative stress biomarker 8-oxodG. Additionally, vitamin C in the blood plasma and inside the cells (leukocytes and prostate tissues) and the expression of *TETs* and *TDG* were assessed.

## Material and methods

### Subjects

The study included two groups of patients: with prostate cancer (n = 71, median age 67 years) and benign prostatic hyperplasia (n = 35, median age 68 years). The control group consisting of 26 healthy men (median age 53 years) without any oncological treatment history was recruited from the national cancer screening program participants. All the study participants, including the healthy subjects, were enrolled in a hospital setting (Jan Biziel University Hospital No 2 in Bydgoszcz, Poland), and none of them were related to one another or had received anticancer therapy before the sample collection.

Peripheral blood samples were taken from all the enrolled subjects. Additionally, the fragments of benign prostatic hyperplasia tissues were obtained from BPH patients, as well as prostate tumors and cancer-free (marginal) prostate tissues from prostate cancer patients. Prostate tissue samples were obtained from prostate biopsy specimens or after surgical resection and were frozen in liquid nitrogen and then stored at −80°C. The biological specimens were collected between May 2020 and July 2022. All the clinical research was conducted pursuant to the provisions of the Declaration of Helsinki. The study protocol was approved by the Bioethics Committee of the Nicolaus Copernicus University in Toruń (No. KB 315/2018). All enrolled participants signed the General Data Protection Regulation and the consent form.

### Methods

Most of the laboratory procedures used in the present study were described in Guz et al. [13]. Additionally, the details concerning gene expression analysis were presented in Linowiecka et al. [16]. Primers and short hydrolysis probes used for the target genes mRNA expression analysis are shown in supplementary data.

### Statistical analyses

The results are presented as median values, interquartile ranges, and 95 % confidence intervals. Statistical analyses were carried out with Statistica 13.3 PL software [TIBCO Software Inc. (2017), version 13. http://statistica.io]. Normal distribution of the study variables was verified with the Kolmogorov–Smirnov test with Lilliefors correction. The variables with non-normal distributions were analyzed with a nonparametric Mann–Whitney U test. The correlations were assessed using the Spearman's correlation analysis. The results were considered statistically significant at *p* < 0.05.

## Results

**Fig. 1:**
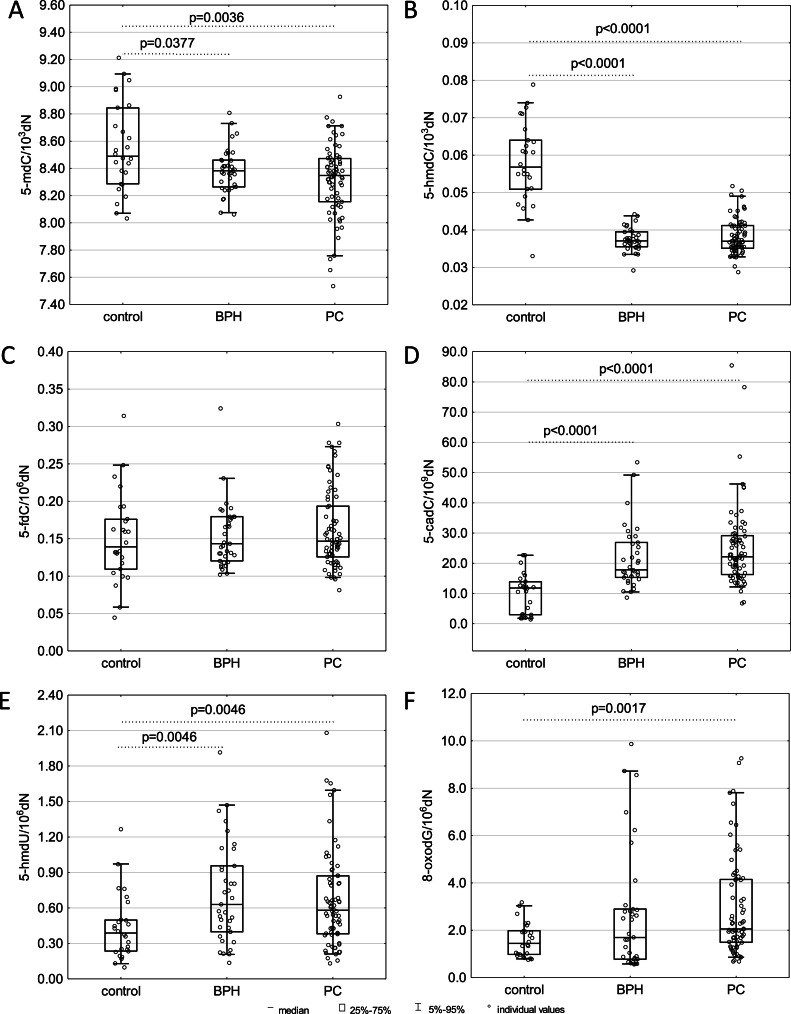
Levels of DNA modifications: (A) 5-mdC, (B) 5-hmdC, (C) 5-fdC, (D) 5-cadC, (E) 5-hmdU, and (F) 8-oxodG in leukocytes’ DNA from healthy controls; patients with benign prostatic hyperplasia (BPH) and prostate cancer (PC). The results were presented as medians, interquartile ranges, and 95 % confidence intervals.

In the present study, the analysis of the levels of 5-mdC and products of the active demethylation process in the genomic DNA from the peripheral blood leukocytes was carried out. Interestingly, a significantly lower content of 5-mdC and 5-hmdC in leukocytes from individuals with BPH (*p* = 0.0377 and *p* < 0.0001, respectively) and PC (p = 0.0036 and *p* < 0.0001, respectively), compared to healthy subjects was observed (Fig. 1A, B). Conversely, higher levels of 5-cadC were detected in leukocytes from patients with PC (*p* < 0.0001) and BPH (*p* < 0.0001) in comparison to the control group. Also, the levels of 5-hmdU in patients with benign prostatic hyperplasia (*p* = 0.0046) and prostate cancer (*p* = 0.0046) were higher than in healthy controls (Fig. 1D, E). Determination of 8-oxodG in leukocytes’ DNA revealed a significantly higher level of this modification in individuals with PC (*p* = 0.0017) in comparison with the control group (Fig. 1F).

**Fig. 2:**
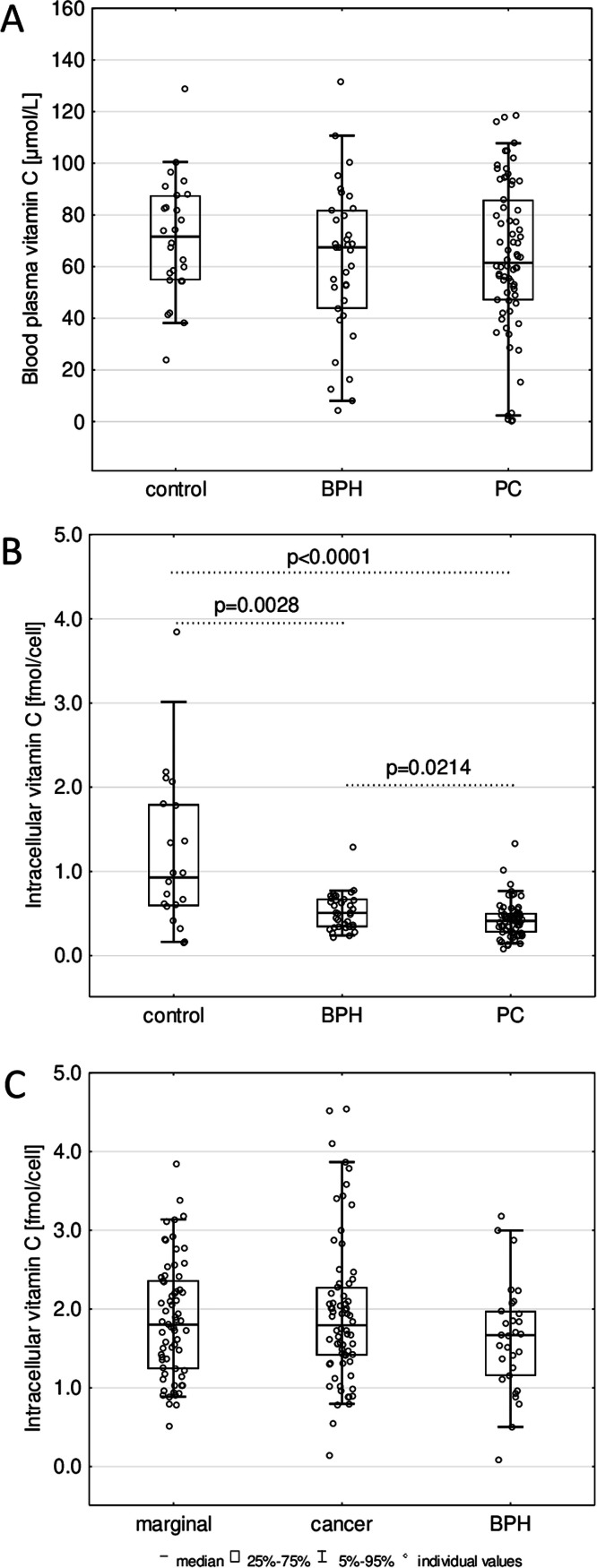
Comparison of vitamin C: (A) in blood plasma of healthy controls, patients with BPH and PC, (B) inside the leukocytes of healthy controls, patients with BPH and PC, (C) in normal/marginal prostate tissues; cancer prostate tissues; and benign prostatic hyperplasia (BPH) tissues. The results were presented as medians, interquartile ranges, and 95 % confidence intervals.

The alterations in the levels of epigenetic modifications in the leukocytes of BPH and PC patients were accompanied by a significantly lower intracellular level of vitamin C (*p* = 0.0028 and *p* < 0.0001, respectively), than in healthy men (Fig. 2B). The median level of vitamin C in blood plasma was 71.577 μmol/L in healthy donors, 61.481 μmol/L in PC patients, and 67.501 μmol/L in BPH patients, respectively. Yet, no significant differences in blood plasma vitamin C concentration between patients and control groups were observed (Fig. 2A).

**Fig. 3:**
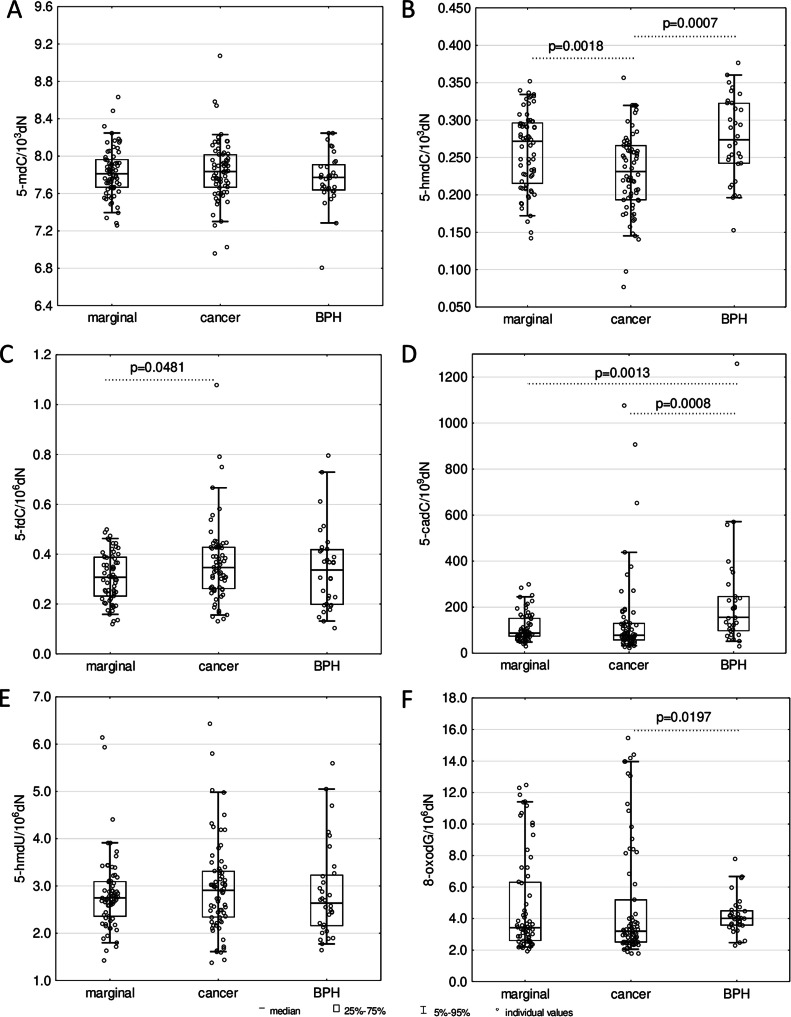
Levels of DNA modifications (A) 5-mdC, (B) 5-hmdC, (C) 5-fdC, (D) 5-cadC, (E) 5-hmdU, and (F) 8-oxodG in normal/marginal prostate tissues; cancer prostate tissues; and benign prostatic hyperplasia (BPH) tissues. The results were presented as medians, interquartile ranges, and 95 % confidence intervals.

**Fig. 4:**
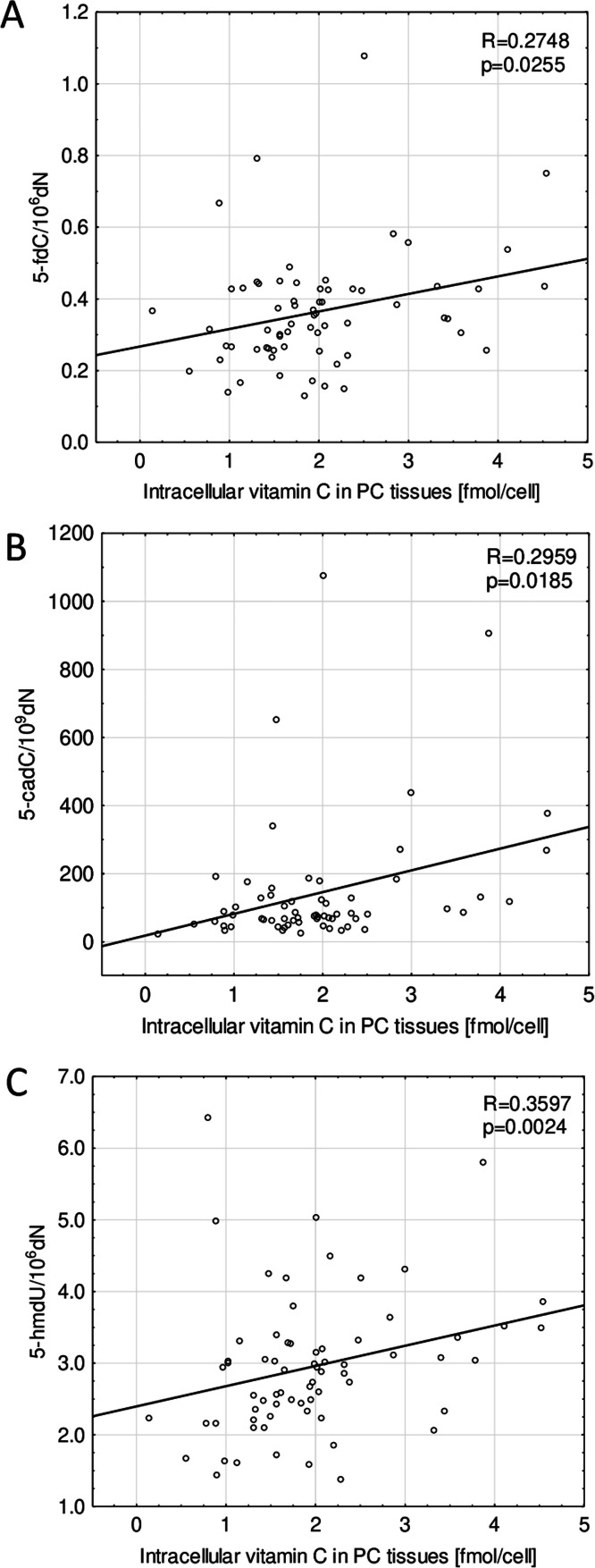
Correlations between the levels of DNA epigenetic modifications (A) 5-fdC, (B) 5-cadC, (C) 5-hmdU, and intracellular vitamin C concentration in cancer prostate tissues.

In prostate tumor tissue samples, a significantly lower level of 5-hmdC opposite to normal/marginal prostate tissues from PC patients (*p* = 0.0018) and BPH tissues (*p* = 0.0007), was noticed. Furthermore, the level of 5-fdC in cancer tissues was significantly higher than in normal prostate tissues (*p* = 0.0481). In turn, hyperplastic prostate tissues were characterized by significantly higher levels of 5-cadC than both, tumors (*p* = 0.0008) and normal/marginal prostatic tissues (*p* = 0.0013). In the BPH tissues, additionally, an increased level of 8-oxodG (*p* = 0.0197) was observed when juxtaposed with tissues taken from PC patients (Fig. 3). Even though no statistically significant between-tissue differences were noticed in intracellular vitamin C concentration (Fig. 2C), significant but weak correlations between 5-fdC (*p* = 0.0255), 5-cadC (*p* = 0.0185), 5-hmdU (*p* = 0.0024), and intracellular vitamin C in prostate cancer tissues were found (Fig. 4).

The levels of DNA modifications (5-hmdC, 5-fdC, 5-cadC, 5-hmdU, 8-oxodG) and intracellular vitamin C in all analyzed prostate tissues were significantly higher (*p* < 0.0001) compared to leukocytes (see supplementary data). Conversely, the level of 5-mdC in DNA isolated from prostate tissues was considerably lower (*p* < 0.0001) than in the DNA of leukocytes (see supplementary data).

**Fig. 5:**
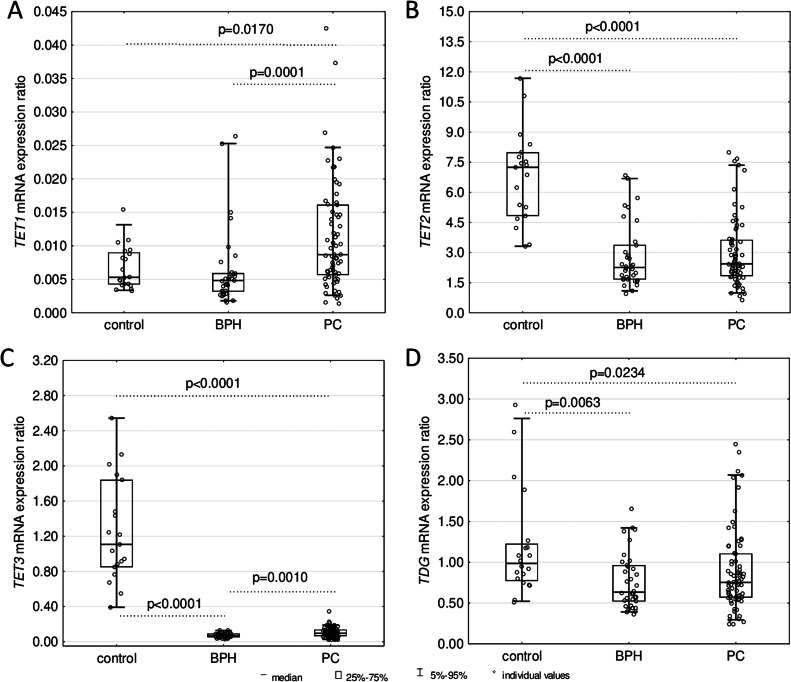
Expressions of (A) *TET1*, (B) *TET2*, (C) *TET3*, and (D) *TDG* mRNA in leukocytes from healthy controls; patients with benign prostatic hyperplasia (BPH) and prostate cancer (PC). The results were presented as medians, interquartile ranges, and 95 % confidence intervals.

**Fig. 6:**
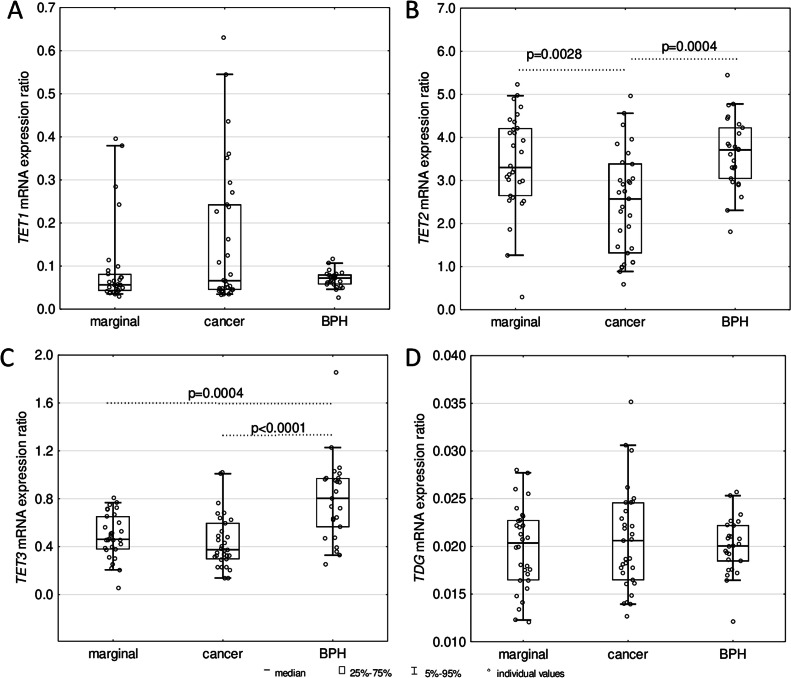
Expressions of (A) *TET1*, (B) *TET2*, (C) *TET3*, and (D) *TDG* mRNA in normal/marginal prostate tissues, cancer prostate tissues, and BPH tissues. The results were presented as medians, interquartile ranges, and 95 % confidence intervals.

The expression level of *TET1* in leukocytes of PC patients was significantly higher compared to controls (*p* = 0.0170) and individuals with BPH (*p* = 0.0001) (Fig. 5A). However, the examined tissues did not exhibit significant differences in *TET1* mRNA expression (Fig. 6A). In turn, the expression of *TET2* and *TET3* in leukocytes of PC (*p* < 0.0001) and BPH patients (*p* < 0.0001) turned out to be significantly weaker than in control (Fig. 5B, C). Similarly, decreased expression of a gene involved in DNA repair – *TDG* was observed in leukocytes of both patient groups (*p* = 0.0063 for BPH and *p* = 0.0234 for PC) compared to healthy subjects (Fig. 5D). Moreover, significantly weaker expressions of *TET2* were found in tumor tissues than in normal/marginal prostate (*p* = 0.0028) and BPH tissues (*p* = 0.0004). Furthermore, *TET3* mRNA expression in PC tissues was decreased compared to BPH tissues (*p* < 0.0001). However, no significant differences were observed regarding *TET1* and *TD*G mRNA levels among the examined tissues (Fig. 6).

## Discussion

Benign prostatic hyperplasia is prevalent among aging males, and PC ranks among the most common male cancers. However, the direct, causal link between these two disorders is still controversial and establishing such a link may be helpful, potentially mitigating the risk of potential progression from BPH to PC. DNA methylation/hydroxymethylation emerges as a key factor in PC development. Of note is that some of the genes exhibiting epigenetic alternation in PC have also been found to be hypermethylated in BPH [17]. Chronic inflammation is another important factor that may be linked with BPH and PC [4,18].

Recent studies have consistently demonstrated decreases in the levels of 5-hmCyt, along with other epigenetic modifications, in various types of human cancers, including PC [19,20]. However, it is largely unknown whether this situation is confined to pathological tissue or may also take place in surrogate tissues such as the leukocytes of patients (see also *Introduction* chapter). Experimental data suggest that a decreased level of the aforementioned modification may be typical of pathological/cancerous tissue or may occur in precancerous injury [21] or conditions that potentially predispose to cancer development [22]. Consequently, this suggests that this process may persist during tumor progression.

Prostate cancer has a good prognosis, with a 5-year survival rate reaching 98 % [23]. However, it remains highly heterogeneous, with patients achieving complete remission but also progressing to metastatic and lethal stages. Therefore, identifying predictive biomarkers for disease progression becomes an important concern. In our study, we analyzed several factors potentially involved in the disease course, including a broad spectrum of DNA epigenetic marks, the expression/activity of TETs and TDG enzymes, as well as the concentration of VC in blood and its intracellular content in both leukocytes and prostate tissues.

The results presented herein demonstrated, for the first time, that the 5-hmdC level in leukocytes was lower in BPH and PC patients compared to healthy controls (Fig. 1B). In turn, PC tissues also presented the lowest level of this modification (Fig. 3B). However, it was associated with global hypomethylation only in leukocytes of both patient groups (Fig. 1A). This indicates that the drop in overall level of 5-hmCyt and 5-mCyt recognized during the possible track of PC development is less pronounced in the malignant tissue than in peripheral blood nuclear cells (PBNCs). This suggests that abnormal DNA methylation may be an integrated process rather than a restricted episode. A significant decrease in 5-mCyt and 5-hmCyt content may be involved in genomic instability, constituting a critical step in PC development. In addition to the lower level of 5-hmdC, leukocytes from BPH and PC patients exhibited increased content of 5-cadC and 5-hmdU (Fig. 1D, E). Among the examined tissues the BPH group contained the highest level of 5-cadC (Fig. 3D). Interestingly, we identified a significant positive correlation between 5-fdC, 5-cadC, 5-hmdU, and iVC levels only in neoplastic tissues of the PC patients group (Fig. 4), providing further evidence for a potential role of VC in shaping epigenetic DNA modifications.

It is possible that the process of iterative DNA oxidation occurring during chronic inflammation, which may induce oxidative stress and malnutrition which may influence VC level, can modulate TET activity, thereby impacting the content of DNA epigenetic modifications (see below).

We observed a highly significant decrease in *TET2* and *TET3* expression in leukocytes of both patient groups (Fig. 5B, C), which may be linked to the observed changes in the modifications of the cellular DNA. While there is no doubt that TET enzymes are directly involved in forming all epigenetic modifications analyzed in this study, still little is known about the factors responsible for this process. Mainly, it is uncertain why the first stage of iterative oxidation stops at the 5-hmCyt step or progresses to the 5-fCyt and 5-caCyt steps. The different affinity of TETs to 5-mCyt, 5-hmCyt, 5-fCyt, and 5-caCyt is one possible explanation (for review, see [24,25]). Another potential explanation is that specific factors recognize the modifications and decide their destiny [26]. The observed increase in *TET1* expression in leukocytes of PC patients (Fig. 5A) may be seen in the context concerning the elevation of 5-cadC and 5-hmdU levels (Fig. 1 D, E), which, in turn, suggests that this enzyme may play a distinctive role in the formation of both these modifications. Of note, recently obtained data identified that c-Myc and Max associated factors, and perhaps also other regulatory proteins, may interact with 5-caCyt but have a weaker affinity for 5-fCyt and show only much less affinity for 5-mCyt and 5-hmCyt [27]. Furthermore, a recent work by Zhou et al. [28] demonstrated that Sall4, an oncogenic protein whose level of expression is very high in PC, correlates with proliferation and metastasis in prostate cancer cells, thereby may further increase TET2-catalyzed oxidation of 5-hmCyt [29].

Of note, we also observed elevated levels of 8-oxodG, a marker of oxidative stress, in the leukocytes of BPH and PC patients and in BPH tissues (Figs. 1F and 3F). It is well-documented that infections and inflammatory conditions are associated with an increase in 8-oxodG levels in tissues, including leukocytes [22,30,31]. The response to inflammation may cause the recruitment of activated leukocytes. This, in turn, may lead to a “respiratory burst” and an increase in oxygen uptake, which results in the extended release of reactive oxygen species, which eventually contributes to oxidative stress and DNA damage [22] (reviewed in [30]).

The endogenous generation of ROS is probably not directly linked to the synthesis of epigenetic DNA modifications [32]. Instead, the pathogenic environment may influence factors responsible for oxidative stress, which in turn may be linked to the generation of epigenetic marks. Indeed, experimental evidence demonstrated that oxidative stress may result in post-translational modulation of TET2 [33]. Moreover, it cannot be excluded that also superoxide, a precursor of ROS, may also play some role in TET-mediated iterative oxidation [34,35].

As mentioned above, the content of another higher-order product of iterative DNA oxidation, namely 5-caCyt, was found to be the highest in leukocytes of the BPH and PC patients and BPH tissues (Figs. 1D and 3D). This phenomenon may be linked to recent data demonstrating that TET2 may yield 5-caCyt without the decrease/changes of 5-hmCyt level [36]. A persistent increase in oxidative stress may possibly influence TET activity (in addition to observed decrease in expression of *TETs*), promoting the increased production of 5-caCyt during the active demethylation process. This evidence, in turn, suggests that oxidative stress may alter the formation of epigenetic DNA modifications. It should be mentioned that this relationship appears to be complicated and its exact course is far from being understood [[33], [34], [35]].

Determination of iVC in human cells often involves inaccurate analyses and post-mortem samples [37]. Reliable assays primarily rely on high-performance liquid chromatography (HPLC)-based techniques, predominantly confined to blood cells [37]. Choosing appropriate normalization methods is also essential. Our study assessed the intracellular concentration of L-ascorbic acid (iVC) using ultra-performance liquid chromatography-tandem mass spectrometry (UPLC-MS/MS), standardized and normalized based on thymine levels [38]. Using this approach, we observed exceptionally high iVC levels in all examined prostatic tissues, regardless of their origin, exceeding those in leukocytes. Typically, VC concentrations are higher in leukocytes than in most human soft tissues [37]. Blood plasma VC levels in patients with BPH and PC were similar to those in healthy individuals (Fig. 2A). While the iVC level was similar in all examined prostatic tissues (Fig. 2C), a gradual and highly significant decrease in its level was observed in leukocytes of BPH and PC patients (Fig. 2B). This phenomenon may be explained in the context of our recently published findings [39]. Under stressful conditions, leukocytes of PC patients exhibit two distinct reactions, both of which correlate with the proposed functions of iVC: as a protective factor safeguarding cellular DNA and/or serving as a reservoir, decreasing VC levels in leukocytes while releasing VC into the plasma to maintain physiological levels. The observed significant decrease in leukocyte iVC levels in BPH and PC patients compared to the control group supports our hypotheses [39].

Several prior studies have shown that VC can enhance the production of 5-hmCyt in cultured cells, likely functioning as a cofactor for TETs during the oxidation of 5-mCyt [10,40]. Recently, we observed a significant increase in 5-hmUra and 5-caCyt levels following ascorbate stimulation [10]. Our recent research [10] confirmed that physiological concentrations of ascorbate maintain stable levels of 5-hmCyt, which is essential for the cell's epigenetic functions. However, much higher concentrations of ascorbate were necessary to achieve a sustained increase in 5-fCyt, 5-caCyt, and 5-hmUra levels, and possibly to initiate the active demethylation process. This observation might indicate cellular adaptation to changing environmental conditions. Although DNA epigenetic patterns in prostate cells are similar to those in other soft tissues, such as the colon [31], their distinctiveness is highlighted by the uniquely high level of 5-hmdU (see supplementary data), a feature not observed in other tissues.

To our knowledge, this is the first study to demonstrate that each group: healthy controls, individuals with BPH, and those with PC exhibits a distinctive pattern of epigenetic modifications in their leukocyte DNA. Consequently, a significant question arises regarding the mechanisms driving the development of these disease-specific epigenetic modification profiles in leukocytes. It is plausible that these profiles result from oxidative stress (variations in redox status) associated with each pathological condition, leading to changes in cellular metabolism and affecting iterative enzymatic DNA modifications. In this context, it is noteworthy that our previous study documented the presence of oxidative stress in leukocytes from patients with colorectal cancer and inflammatory bowel disease, including polyps [22,31].

Experimental studies suggest TET enzymes might be involved in creating 5-hmUra, a molecule influencing gene expression. Moreover, 5-hmUra in DNA appears to interact with proteins that remodel chromatin and regulate transcription [8]. Interestingly, 5-hmUra similar to 5-fCyt might have its own regulatory functions acting as a signal molecule for "poised"/bivalent genes. Notably, this modification was shown to act as a transcription regulator [41,42]. Moreover, a BER enzyme TDG recognizes 5-hmUra [43] and may regulate transcription independently of its repair activity. Despite these findings, 5-hmUra levels in various cells remain relatively stable, similar to those seen in leukocytes, about 0.4/10^6^dN in human colorectal cancer [22] and animal tissues [44]. Given these characteristics of 5-hmUra, its significant increase in prostate cells might be directly linked to chromatin reorganization, a trait characteristic of the prostate epithelium's regenerative capacity [45,46]. This trait might also be associated with androgen metabolism and its role in the pathogenesis of prostate cancer (PC) and benign prostatic hyperplasia (BPH) [18]. It is hypothesized [46] that the prostate epithelium might contain stem cells responsible for tissue regeneration [46]. Interestingly, hematopoietic stem cells presented with a very high concentration of iVC [47], close to that in our findings regarding prostatic cells.

All these factors could change TET activity in patients with specific pathologies, potentially leading to the formation of disease-specific epigenomes. A pertinent question arises regarding how prostate function may be linked with the characteristic levels of epigenetic modifications in PBNCs. As previously mentioned, its formation through TET activity can be stimulated by vitamin C [48]. The progressive decrease of intracellular ascorbate in the blood leukocyte (from control to BPH and PC) may be linked to the observed changes in epigenetic DNA modification.

## Conclusions

Various evidence indicates that BPH and PC share some molecular and pathologic links on top of the previously established epidemiologic relationship between these diseases. However, whether BPH directly causes PC or if there are random risk factors shared between both conditions, remains largely unknown [4,18]. Notably, the very convincing and intriguing data from Orsted et al. [4], comprising about 3,000,000 men, demonstrated that BPH was linked to an increased risk of PC development and mortality.

Several works demonstrated that inflammation is a major causative factor in both pathologies BPH and PC [4,18]. As mentioned in the previous chapter, other key factors involved in PC development are DNA methylation and hydroxymethylation (reviewed in [19,20,49]).

Our studies clearly show unique changes in several parameters, associated with inflammation and epigenetic DNA modifications, both in BPH and PC cells and in leukocytes, in comparison to healthy/control samples.

In our previous papers, similarly to the present work, we discussed epigenetic DNA modifications and other factors linked with chronic inflammation in colon cancer and the conditions that predispose to its development (colon adenomas and inflammatory bowel disease) to ascertain their influence on carcinogenesis. It was demonstrated that each condition exhibited characteristic patterns of DNA modifications, distinguishing them from one another and from the healthy colons. Therefore, the findings described herein, namely the changes in 5-mCyt, 5-hmCyt, 5-caCyt, 5-hmUra, and iVC in leukocytes may serve as causal markers indicating the first stages of prostate carcinogenesis.

Intriguingly, the majority of key parameters analyzed herein were more pronounced in leukocyte fraction than in the prostate tissues. A partial explanation of this phenomenon may be linked to the involvement of leukocytes/neutrophils in prostate carcinogenesis. Neutrophils constitute up to 70 % of all leukocytes circulating in the blood [50]. There is increasing evidence that blood neutrophils actively participate in various aspects of carcinogenesis [51], including prostate cancer [52]. Moreover, neutrophils serve as indicators of cancer-related systemic inflammation [53]. Importantly, a fraction of neutrophils recruited to the pathological tissues can migrate back into the circulation/blood [54], and in this way, the observed alterations may reflect dynamic changes associated with PC development.

Summing up, characteristic DNA methylation/hydroxymethylation and iVC profiles are more pronounced in PBNC fraction of BPH/PC blood patients than in solid tissues, suggesting their potential utility as early noninvasive markers of prostate cancer development.

## Declarations of competing interest

The authors declare that they have no known competing financial interests or personal relationships that could have appeared to influence the work reported in this paper.

## Data availability statement

The data presented in this study is available on request from the corresponding author.

## Disclosure of using AI-assisted technologies

During the preparation of this work, the authors used Grammarly (v. 1.0.37.777) and ChatGPT-4o in order to improve language and readability. After using this tool, the authors reviewed and edited the content as needed, and take full responsibility for the content of the publication.

## CRediT authorship contribution statement

**Jolanta Guz:** Writing – review & editing, Writing – original draft, Visualization, Resources, Investigation, Formal analysis, Conceptualization. **Ewelina Zarakowska:** Writing – review & editing, Writing – original draft, Visualization, Resources, Investigation, Conceptualization. **Pawel Mijewski:** Investigation. **Aleksandra Wasilow:** Investigation. **Fabian Lesniewski:** Investigation. **Marek Foksinski:** Project administration. **Bartosz Brzoszczyk:** Resources. **Piotr Jarzemski:** Resources. **Daniel Gackowski:** Writing – review & editing, Writing – original draft, Visualization, Supervision, Resources, Investigation, Conceptualization. **Ryszard Olinski:** Writing – review & editing, Writing – original draft, Visualization, Supervision, Funding acquisition, Conceptualization.

## Declaration of competing interest

The authors declare that they have no known competing financial interests or personal relationships that could have appeared to influence the work reported in this paper.

## Appendix Supplementary materials

Image, application 1
