# Supplementary material for: Epigenetic DNA modifications and vitamin C in prostate cancer and benign prostatic hyperplasia: Exploring similarities, disparities, and pathogenic implications
Source: Neoplasia. 2024 Oct 29;58:101079. doi: 10.1016/j.neo.2024.101079 (PMC11550371; doi:10.1016/j.neo.2024.101079)
Supplement: Supplementary file 1 [file mmc1.docx]

**Supplementary data**

**Detailed results of the performed analyses**

Table 1. Levels of active demethylation products, 8-oxodG, and intracellular vitamin C in leukocytes from healthy men (control); patients with benign prostatic hyperplasia (BPH) and prostate cancer (PC). Values are expressed as median and interquartile range.

|  | **Control** | **BPH** | **Prostate Cancer** |
| --- | --- | --- | --- |
| **5-mdC/10^3^dN** | 8.489 (8.286-8.844) | 8.382 (8.264-8.461) | 8.348 (8.155-8.472) |
| **5-hmdC/10^3^dN** | 0.057 (0.051-0.064) | 0.037 (0.035-0.039) | 0.037 (0.035-0.041) |
| **5-fdC/10^6^dN** | 0.139 (0.109-0.176) | 0.143 (0.121-0.179) | 0.147 (0.126-0.193) |
| **5-cadC/10^9^dN** | 11.801 (2.982-13.886) | 17.838 (15.327-26.889) | 22.152 (16.273-29.154 |
| **5-hmdU/10^6^dN** | 0.388 (0.235-0.498) | 0.630 (0.398-0.956) | 0.581 (0.381-0.871) |
| **8-oxodG/10^6^dN** | 1.445 (0.977-1.978) | 1.690 (0.775-2.896) | 2.053 (1.491-4.145) |
| **Intracellular vitamin** [fmol/cell] | 0.927 (0.594-1.792) | 0.506 (0.344-0.673) | 0.412 (0.279-0.501) |

Table 2. Levels of active demethylation products, 8-oxodG, and intracellular vitamin C in normal/marginal prostate tissues, cancer prostate tissues, and benign prostatic hyperplasia (BPH) tissues. Values are expressed as median and interquartile range.

|  | **Normal marginal prostate tissues** | **Cancer prostate tissues** | **BPH tissues** |
| --- | --- | --- | --- |
| **5-mdC/10^3^dN** | 7.811 (7.665-7.966) | 7.835 (7.664-8.016) | 7.772 (7.637-7.910) |
| **5-hmdC/10^3^dN** | 0.272 (0.215-0.297) | 0.231 (0.193-0.266) | 0.274 (0.242-0.323) |
| **5-fdC/10^6^dN** | 0.307 (0.232-0.388) | 0.346 (0.260-0.428 | 0.336 (0.198-0.419 |
| **5-cadC/10^9^dN** | 86.986 (73.073-152.909) | 77.742 (56.753-130.107) | 155.653 (96.101-247.327) |
| **5-hmdU/10^6^dN** | 2.748 (2.358-3.099) | 2.908 (2.338-3.315) | 2.637 (2.156-3.234) |
| **8-oxodG/10^6^dN** | 3.424 (2.584-6.326) | 3.200 (2.511-5.193) | 4.012 (3.579-4.499) |
| **Intracellular vitamin** [fmol/cell] | 1.802 (1.244-2.362) | 1.796 (1.417-2.276) | 1.668 (1.154-1.969) |

Table 3. Comparison of active demethylation products, 8-oxodG, and intracellular vitamin C between leukocytes and prostate tissues

|  | **PC leukocytes vs. marginal prostate tissues**  ***p-value*** | **PC leukocytes vs. cancer prostate tissues**  ***p-value*** | **BPH leukocytes vs. BPH tissues**  ***p-value*** |
| --- | --- | --- | --- |
| **5-mdC/10^3^dN** | p<0.0001 | p<0.0001 | p<0.0001 |
| **5-hmdC/10^3^dN** | p<0.0001 | p<0.0001 | p<0.0001 |
| **5-fdC/10^6^dN** | p<0.0001 | p<0.0001 | p<0.0001 |
| **5-cadC/10^9^dN** | p<0.0001 | p<0.0001 | p<0.0001 |
| **5-hmdU/10^6^dN** | p<0.0001 | p<0.0001 | p<0.0001 |
| **8-oxodG/10^6^dN** | p<0.0001 | p<0.0001 | p<0.0001 |
| **Intracellular vitamin C** [fmol/cell] | p<0.0001 | p<0.0001 | p<0.0001 |

Table 4. Expression of *TETs* and *TDG* mRNA in leukocytes from healthy men (control); patients with benign prostatic hyperplasia (BPH) and prostate cancer (PC). Expression levels were presented as relative values normalized to reference genes (*HMBS*, *TBP,* and *G6PD*). Values are expressed as median and interquartile range.

|  | **Control** | **BPH** | **Prostate Cancer** |
| --- | --- | --- | --- |
| ***TET1*** | 0.0053 (0.0042-0.0090) | 0.0048 (0.0032-0.0059) | 0.0087 (0.0057-0.0161) |
| ***TET2*** | 7.243 (4.836-7.978) | 2.257 (1.670-3.379) | 2.435 (1.842-3.628) |
| ***TET3*** | 1.108 (0.850-1.839) | 0.066 (0.056-0.091) | 0.096 (0.068-0.137) |
| ***TDG*** | 0.985 (0.774-1.223) | 0.634 (0.521-0.961) | 0.753 (0.570-1.106) |

Table 5. Expression of TETs and TDG mRNA in normal/marginal prostate tissues, cancer prostate tissues, and benign prostatic hyperplasia (BPH) tissues. Expression levels were presented as relative values normalized to reference genes (*HMBS, TBP,* and *G6PD*). Values are expressed as median and interquartile range.

|  | **Normal marginal prostate tissues** | **Cancer prostate tissues** | **BPH tissues** |
| --- | --- | --- | --- |
| ***TET1*** | 0.057 (0.043-0.081) | 0.066 (0.045-0.242) | 0.072 (0.058-0.079) |
| ***TET2*** | 3.300 (2.642-4.214) | 2.570 (1.319-3.385) | 3.710 (3.042-4.228) |
| ***TET3*** | 0.461 (0.378-0.652) | 0.374 (0.296-0.598) | 0.804 (0.565-0.972) |
| ***TDG*** | 0.020 (0.016-0.023) | 0.021 (0.016-0.025) | 0.020 (0.018-0.022) |

**The primer sequences and numbers of UPL probes used for the target gene mRNA expression analysis.**

Table 6. Primers and short hydrolysis probes used for the target gene mRNA expression analysis.

| **Gene** | **Forward primer sequence** | **Reverse primer sequence** | **UPL** |
| --- | --- | --- | --- |
| ***TET1*** | 5’-TCTGTTGTTGTGCCTCTGGA-3’ | 5’-GCCTTTAAAACTTTGGGCTTC-3’ | #57 |
| ***TET2*** | 5’-GCCTTTGCTCCTGTTGAGTT-3’ | 5’-ACAAGGCTGCCCTCTAGTTG-3’ | #38 |
| ***TET3*** | 5’-CACTCCGGAGAAGATCAAGC-3’ | 5’-GGACAATCCACCCTTCAGAG-3’ | #1 |
| ***TDG*** | 5’-GAATGGAAGCGGAGAACG-3’ | 5’-TTGCTGTTCATTCACAACTGC-3’ | #41 |
